# A case of congenital fibular hemimelia associated with skeletal and non-skeletal malformations

**DOI:** 10.1093/bjrcr/uaaf008

**Published:** 2025-02-18

**Authors:** Mahmoud R Manasra, Rahaf E Farah, Roua E Farah, Sama S Yassin, Shadi A Abuisneina

**Affiliations:** Palestine Polytechnic University College of Medicine and Health Science, Hebron, P175, Palestine; Palestine Polytechnic University College of Medicine and Health Science, Hebron, P175, Palestine; Palestine Polytechnic University College of Medicine and Health Science, Hebron, P175, Palestine; Palestine Polytechnic University College of Medicine and Health Science, Hebron, P175, Palestine; Palestine Polytechnic University College of Medicine and Health Science, Hebron, P175, Palestine

**Keywords:** fibular hemimelia, ectromelia, prenatal diagnosis, spina bifida, case report

## Abstract

Fibular hemimelia (FH) is a rare congenital abnormality where the fibula is either totally or partially absent. It can occur alone or alongside other skeletal malformations, and in very few cases, it may occur along with non-skeletal anomalies. A 4-year-old female was diagnosed with unilateral right-sided FH, accompanied by limb shortening, a right-side ankle deformity, valgus foot, and 3 lateral rays that had been totally absent since the first week of birth. And she was incidentally diagnosed with spina bifida occulta at a 3-year-old age. FH is most commonly unilateral and mostly affects the right side, leads to a limb-length discrepancy, and maybe comes as a symptom of a syndrome such as Foetus-Fibula-Ulna syndrome and so on. Risk factors include prenatal history, drugs, and no supplementation intake. Together, these elements could be a contributing factor to our condition. The congenital limb abnormalities may be discovered during pregnancy by sonography. If present, other investigations need to be done to differentiate the diagnosis. Treatment according to degree: mild, moderate, and severe cases. In our case, the type 2 FH characteristic was shown by sonography, accompanied by limb shortening, lateral rays absent, and a non-skeletal anomaly (spina bifida). These anomalies very rarely come with each other at the same time. To the best of our knowledge, this case is exceptional in that FH is present at birth alongside spina bifida.

## Introduction

Fibular hemimelia (FH) is a rare congenital limb malformation ranging from complete to partial absence of the fibula bone.^1^ With an incidence of 1 in 40 000 live births. It may occur as an isolated anomaly or as part of a skeletal malformation syndrome such as limb shortening, absent lateral rays of the foot, clubfoot, tarsal coalition, and hypoplasia of the lateral femoral condyle with knee valgus.^2^^,^^3^

Very rarely, FH is combined with non-skeletal malformations such as anterior chamber anomalies or anophthalmia, cardiac anomalies, renal dysplasia, thrombocytopenia, thoracoabdominal schisis, and spina bifida.^4^

The exact cause of FH is unknown. A spontaneous genetic error occurs during limb bud development at 6 to 8 weeks after conception.^3^

We present a case of type 2 right FH according to the Achterman and Kalamchi classification with both skeletal deformities, which are absent lateral rays and limb shortening by 15 cm, and a non-skeletal malformation, which is spina bifida, in a 4-year-old female. Also, we discussed risk factors and diagnosis.

This work has been reported in line with SCARE criteria, which are used by authors, journal editors, and reviewers to increase the robustness and transparency of reporting surgical cases.^5^^,^^6^

## Case presentation

A 4-year-old female was presented to the outpatient clinic by her mother when she was 7 days old because the mother noticed that the patient’s right leg was shorter than the left and absent of the 3 lateral fingers. She was born at 36 + 4 weeks by caesarian section because of a foetal breech presentation to a non-consanguineous 20-year-old mother (G2P1Ab1) and a 34-year-old father. During the first trimester of pregnancy, the mother took ceftriaxone, metronidazole (category B), glycerine suppository (category C), and exploratory laparotomy to rule out heterotopic pregnancy. She did not take folic acid or any other vitamin supplements, and she had no history of radiation exposure during this period.

On examination of the patient, the right lower limb was shorter by 15 cm than the left lower limb, and both thighs were normal and equal in length without any bowing. Right valgus foot and ankle deformity, complete absence of 3 lateral rays, and overlapping between the first and second toes. The left leg and ankle were normal without any rays. The upper limbs were bilaterally symmetrical without bowing or digital anomalies. There were no facial dysmorphic features. Cardiac and respiratory examinations reveal no heart murmurs and normal breath sounds. Ophthalmic and female genital examinations were normal. There was no organomegaly. A clinical examination of the parents did not reveal any limb or finger anomalies.

**Figure 1.**
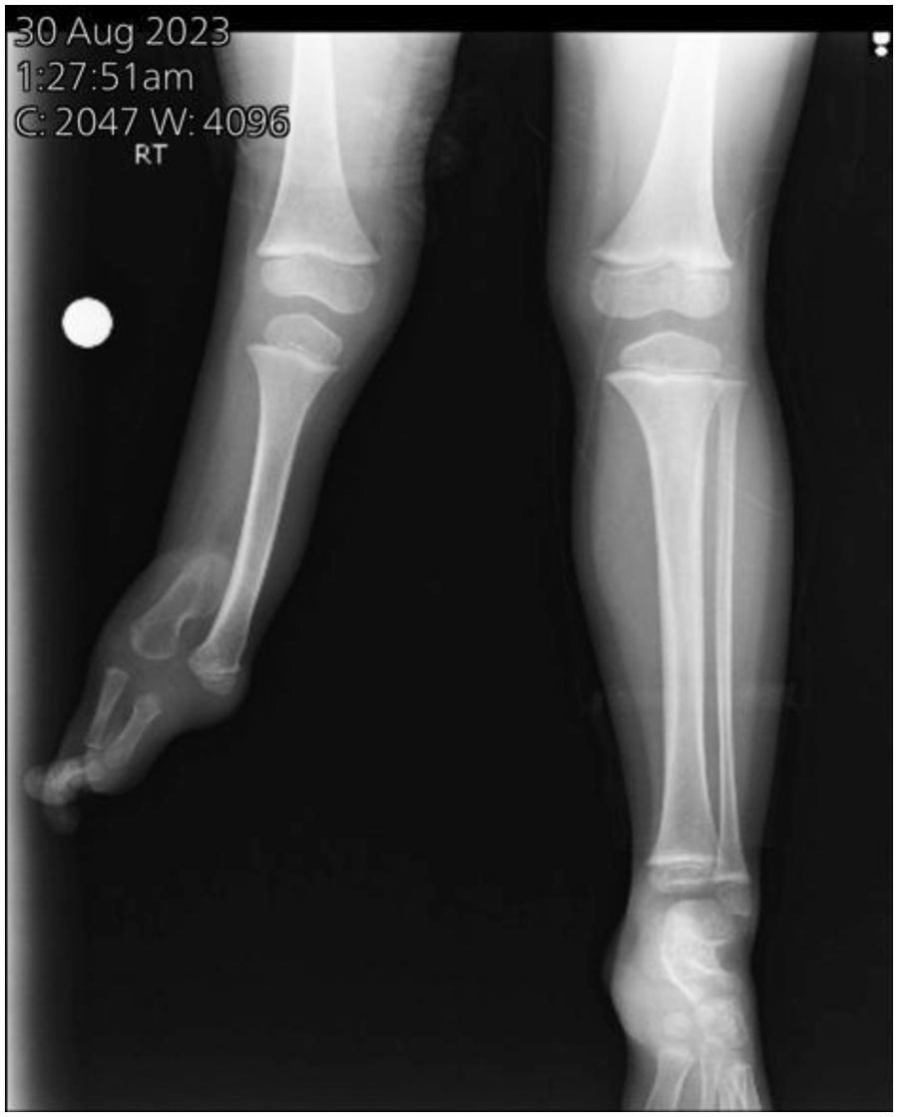
Plain radiograph: anteroposterior view showing complete absence of right fibula and absence of 3 lateral rays.

**Figure 2.**
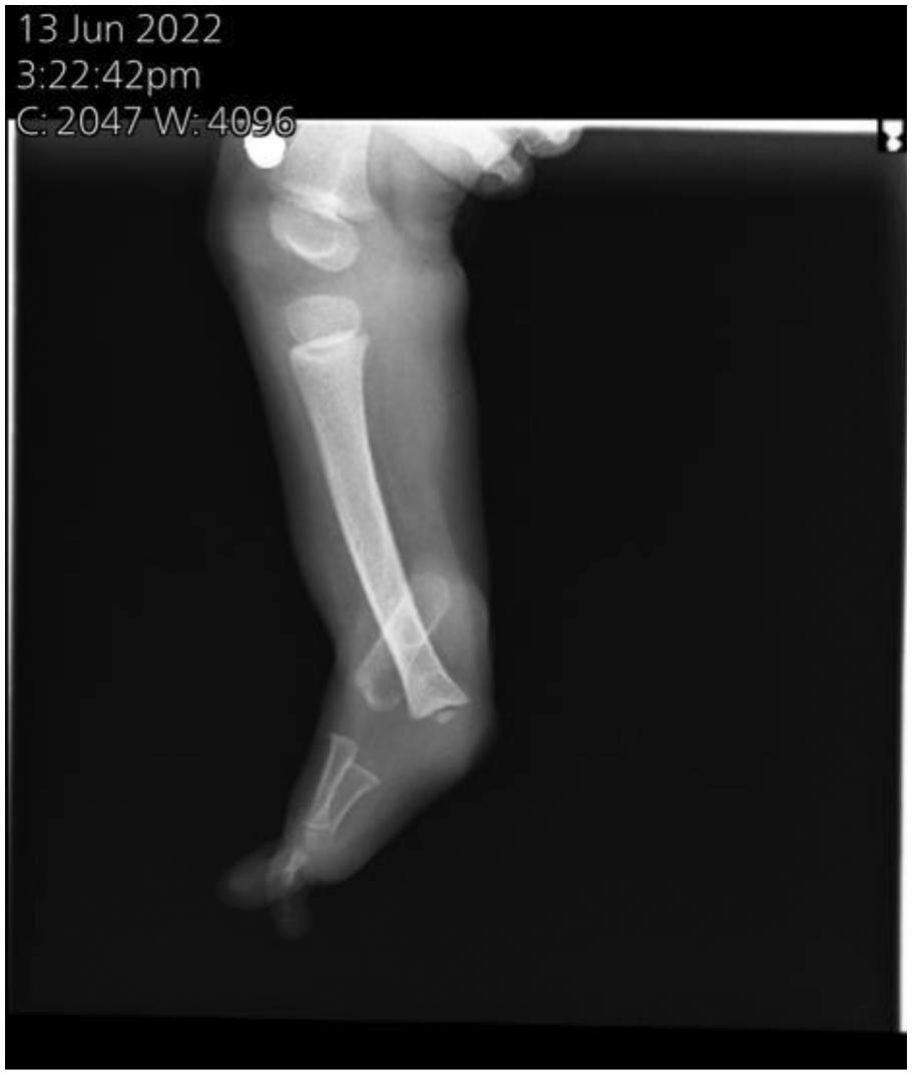
Plain radiograph: lateral view showing complete absence of right fibula and absence of 3 lateral rays.

Anteroposterior and lateral radiographs were done. It showed the complete absence of the right fibula and the absence of 3 lateral rays (Figures 1 and 2).

**Figure 3.**
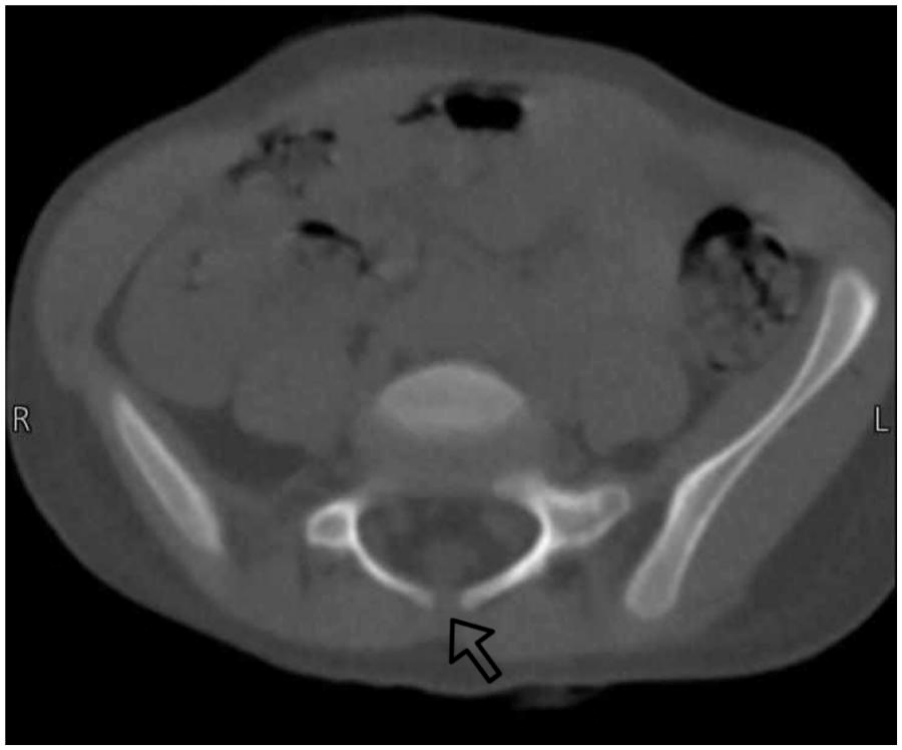
Axial CT scan: showing defect in spinous process (black arrow) at the level of the lumbar spine (L5).

At the age of 3 years, the patient presented to the emergency department due to recurrent vomiting that was non-bloody and non-mucus, associated with poor feeding and hypoactivity. Several ultrasounds were done during this year to rule out intussusception, but there was no sonographic sign of intussusception. A CT was done to rule out intestinal mass because the patient had multiple enlarged lymph nodes on ultrasound. A CT scan without contrast was done to rule out mesenteric lymphadenitis and other abnormalities. Incidentally, spina bifida occulta at level L5 and sacral vertebral measuring 5 mm in size were found (Figure 3).

## Discussion

**Figure 4.**
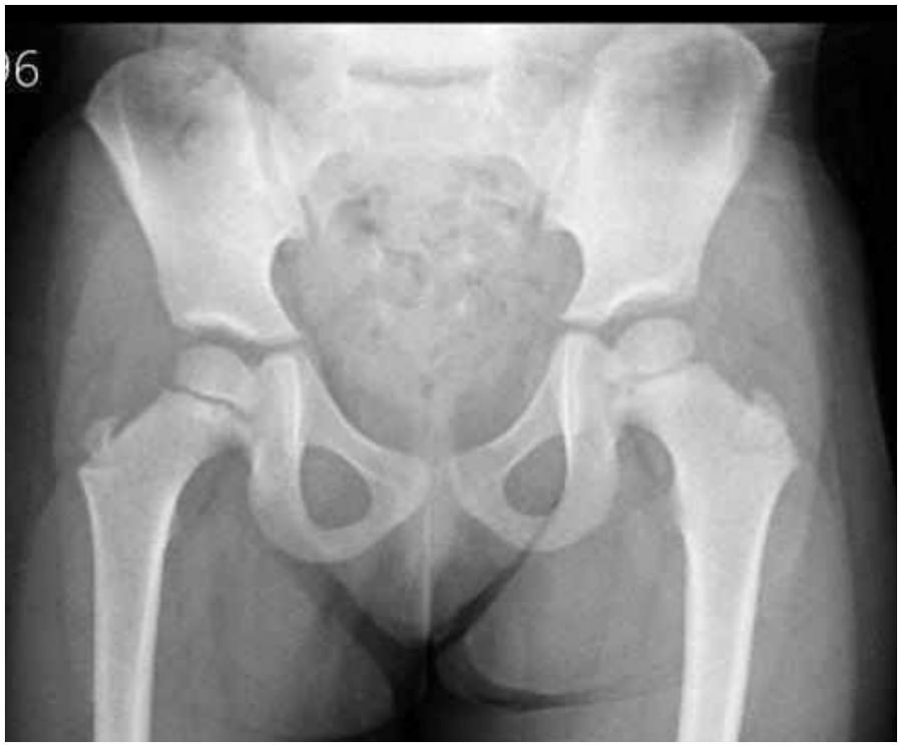
Hip radiograph: anteroposterior view showing no femur abnormalities.

FH is an uncommon longitudinal deficit characterized by a shortening or lack of fibula. Fibula can range from a little deficit to non-existent. Fetus-Fibula-Ulna syndrome, congenital short tibia with missing or dysplastic fibula, congenital deficit of proximal femoral focal deficiency, and intercalary hemimelia of the fibula can also occur with FH. Due to a folate deficit, our case is unique in that it combines FH with spina bifida. Significant shortening of the femur, ossification, shortened or anteriorly bent tibia, malformed or missing fibula with normal mineralization, and foot abnormalities are the primary sonographic findings.^7^ These findings are consistent with our case except for the femur finding, which is normal. About 60%-80% of instances are unilateral, and the right side is afflicted more frequently than the left.^8^ At the end of the 8th or 10th week of pregnancy, the upper and lower limbs’ embryologic growth and recording by sonography occur, but because of negligence, our patient’s mother did not do any antenatal screening, including sonography. Limb development requires the interplay of several complex regulatory proteins, including hedgehog proteins, homeobox factors, fibroblast growth factor, and bone morphogenic proteins.^9^ Although the exact cause is uncertain, it is hypothesized that disturbance of blood flow and muscle development leads to disruption of vascular development.^10^ Following a diagnosis of congenital limb deficit, a comprehensive anatomical scan for further system defects should be performed on the foetus. Although longitudinal limb abnormalities can happen on their own, they can also be a symptom of another condition. To determine whether there is a family history of limb abnormalities, the parents should be questioned and checked for them. Our patient denies any history of limb abnormalities. Identification of etiologic variables may be aided by a comprehensive pregnancy history that includes drugs, medicines, trauma, diabetes mellitus, exposure to viruses, drug usage, and chorion villus samples in the early weeks of pregnancy. As we mentioned earlier, our patient mother took unsafe medications, which are metronidazole (category B) and glycerine suppository (category C). Foetal anatomic scanning can be done using 2D and 3D ultrasonography for the differential diagnosis of FH,^9^^,^^10^ although our case did not do detailed ultrasonography or other antinatal screening, probably due to a history of infertility or negligence. All foetal long bones should be measured once long-bone shortening has been established. Our measurements showed that the right lower limb was shorter by 15 cm than the left lower limb, and both thighs were normal and equal in length without any bowing. To identify any co-existing disorders, assessments of the foetal facial profile, cardiovascular, neurological, genitourinary, and gastrointestinal systems must also be carried out, which was all normal in our case. Making a care plan after a presumptive diagnosis requires early examination using a multidisciplinary approach involving a paediatric orthopaedic surgery team and a geneticist.^11^ When diagnosing and assessing the severity of an illness, imaging is crucial in guiding its care. A hypoplastic or aplastic fibula can be seen on radiographs of the knee and foot. Other features include a shallow trochlear groove, a hypoplastic lateral femoral condyle, and either an absence or a hypoplastic intercondylar notch. Usually convex, the proximal tibial epiphysis lacks or has hypoplastic tibial spines. Recognized characteristics also include genu valgum deformity and tibial bending.^12^ In order to rule out proximal focal femoral deficits, a hip radiograph should also be obtained.^13^ Our patient’s hip radiograph shows no proximal femoral deficit, and the femur was generally normal (Figure 4). For FH, a number of categorization schemes have been developed; these schemes can support judgements about surgical technique and parental counselling. In the postnatal phase, the Achtermann and Kalamchi categorization systems are more frequently employed.^7^^,^^9^ However, there are 3 primary forms of fibula absence: type 1 (10% of cases) is marked by a moderate-to-non-existent bent tibia and whole or partial loss of the fibula. Characterized by unilateral fibula absence, anterior tibial bending, and severe limb shortening, type 2 accounts for 35% of all cases. Cases with same-leg and foot abnormalities and unilateral or bilateral fibula absences are included in type 3 (55% of all cases).^10^ Our case was diagnosed as type 2 FH based on our radiograph findings, which included a bent tibia, valgus deformity, and lack of the right fibula. The degree of fibular insufficiency, related femoral abnormalities, and foot, ankle, or knee deformities all affect the prognosis of FH.^7^ Our patient had a good prognosis, as she did not have any bad prognostic factors, as mentioned above. The degree of leg-length discrepancy and foot function are the determining factors in the treatment of FH. A limb lengthening procedure is advised for patients with mild-to-moderate foot deformities who also have stable hip, knee, and ankle.^14^ Promising results have been seen with the implementation of Ilizarov’s limb-lengthening method, including substantial limb lengthening and foot preservation.^15^ Amputation is the suggested course of treatment for severe foot malformations (Syme’s and Boyd’s),^14^ but the family refuses to follow this recommendation. Changulani et al^15^ state that the quantity of radiation in the foot can be used to guide therapy and determine the severity of the deformity. Conversely, children with fewer than 3 rays required Syme’s amputation.^15^ Every case is treated differently and is completed at institutions that have access to a multidisciplinary team consisting of paediatricians, physical therapists, and orthopaedists, as well as a wealth of information. Children with FH who get any of the 2 treatment options have a lower quality of life, despite the fact that there is no connection between FH patients and mental disability.^11^

## Learning points

- When it comes to directing orthopaedic care in patients with fibrular hemimelia, a radiologist must be knowledgeable of the characteristic that is linked to a variety of problems in the knee joint.
- Patients with fibular hemimelia should undergo a full history and physical examination to detect skeletal and non-skeletal abnormalities (eg, spina bifida).
- It is impossible to overstate the value of a comprehensive previous medical and surgical history in assisting with the diagnosis of some complicated illnesses.
